# Supplementary material for: Association of exogenous factors with the access to innovative pharmaceutical products in Hungary
Source: PLoS One. 2023 Feb 6;18(2):e0281280. doi: 10.1371/journal.pone.0281280 (PMC9901764; doi:10.1371/journal.pone.0281280)
Supplement: Attachment — Submitted filename: Response.pdf [file pone.0281280.s001.pdf]

Submission ID: PONE-D-21-40982
Response to the Reviewer

Dear Editor,

On behalf of all the authors I would like to thank you for giving us the opportunity to submit a revised draft of the manuscript „*Association of Exogenous Factors with the Access to Innovative Pharmaceutical Products in Hungary*" for publication in PLOS One. We appreciate the time and effort that you and the reviewer dedicated to providing feedback on our manuscript and are grateful for the insightful comments on and valuable improvements to our paper.

We have incorporated most of the suggestions made by the reviewer and carried out some minor adjustments as well. Those changes are carried out with tracked changes in the manuscript, and as requested, a clean copy is provided. Please see below, **in blue**, for a point-by-point response to the reviewers' comments and concerns. All row numbers refer to the revised manuscript file with tracked changes.

Yours sincerely,

Gergő Merész

Comments from the editor, reviewers and point – to – point answer

Reviewer 1:

General comments:

1. My main reservations rest with a) the lacking description of the decision-making process in Hungary that would elucidate what could potentially be changed and how;

   Our response:
   Thank you! A concise description has been added on the decision making process (rows 60-79) and the potential changes that may be carried out based on lessons learned from this research (rows 305-317).

2. (...) b) the partial contextualisation within the broader literature on determinants of coverage decisions;

   Our response:
   Thank you! We have elaborated the text to provide a better description potential determinants of coverage decisions using a review on 6 European countries (rows 262-268).

3. (...) and c) the need for a more nuanced discussion of the lessons learned (is the article really advocating for managed entry agreements for all new medicines? at least the issue of administrative burden, transparency and cost containment should be discussed).

   Our response:
   Thank you! The relevant parts of the manuscript where the lessons learned are addressed has been updated (abstract: rows 41-50, discussion: rows 327-329) as a response to the issues highlighted.

<u>Comments on specific sections:</u>

1. Abstract: The independent variables need to be more clearly named. The "need for legilative change to reimburse" is not clear without the context of the Hungarian reality that only comes in the main text. Part of the conclusions (the recommendaitons) does not appear equivalently in the main text. The recommendations should consider feasibility (see general comments above).

   Our response:
   Thank you! The introduction (rows 22-29) and conclusion (rows 41-48) paragraphs of the abstract has been amended to better reflect on these observations.

2. Introduction: When references to the literature are made (e.g. lines 46-61), it is not clear if you refer to all literature examining the relation between endogenous/exogenous factors and reimbursement decisions or only literature on Hungary. This is important to do very clearly, because there is quite a bit of research on what influences reimbursement decisions in different health systems, which is not captured in the introduction but is important for context. Please add a more comprehensive summary of available evidence (in general and in HUngary in particular) to also help set up a discussion of what this study adds later in the manuscript.

   Our response:
   Thank you! The corresponding lines and the paragraph between lines 80-89 has been updated to reflect on these observations. However, we would like to emphasize that this study evaluates the effect of potential determinants to coverage decisions that are only anecdotally described.

3. Introduction: Lines 67-71: how is this different from the distinction you make in lines 62-67? The existence of RSA?

   Our response:
   Thank you! We have added further explanation in rows 106-107 and 110-117 to make a better distinction between these cases.

4. Introduction: Lines 76-77: please justify the assessment of the usefulness of IFR (you will need to explain better what they are and how they fit in the HUngarian reimbursement system)

   Our response:
   Thank you! We have added further explanation in rows 123-133 for the role of IFRs in the local reimbursement system.

5. Generally for the introduction: Please provide an overall description of how reimbursement of new mediicnes works in Hungary (one paragraph, not very long) that you can use as a basis for details brought up and used in the methodology (such as need for legislative change or IFRs). This is necessary for understanding your paper and how it fits/what it adds better. You could also add a figure if helpful.

   Our response:
   Thank you! We feel that this observation has been addressed by our reply on the first (a) general observation of the Reviewer.

6. Methods: Generally, information on Managed Entry Agreements is not publicly available, but you seem to have at least some in Hungary. Please makes this clear early on tin the methods.

   Our response:
   Thank you! We clarified the availability of information on RSAs in rows 158-159.

7. Lines 107-116: please check redundancy with the introduction. If you end up providing a foundational description as suggested above, you can be shorter in the methods when it comes to the definition of your variables.

   Our response:
   Thank you! We provided the suggested description, and the respective paragraph was updated.

8. Lines 123-124: this is not the place for a funding statement of your work, should be moved to the peripherals of the manuscript.

   Our response:
   Thank you! We have removed this sentence.

9. Lines 144-145 "however, this variable was recorded...200 million HUFs". This needs further explanation.

   Our response:
   Thank you! Rows 198-201 were adjusted to better explain how the expenditure on IFRs were handled.

10. Line 152: "changing the reimbursement technique" - what does this mean? might be solved with a better description of processes in the intro as suggested above.

    Our response:
    Thank you! The wording in rows 115-117, 208 were adjusted for more clarity.

11. <u>Results</u>: Lines 188-192: the logic of what is in the table and what is reported only is not always clear. Is the year of submission an independent variable?

    Our response:
    Thank you! Rows 243-244, 246 were adjusted for more clarity.

12. Discussion: Lines 198-200: you should not bring in new potential independent variables to discard at this point, this should be done earlier. Also perhaps spend a few words in the introduction to discuss how you categorise endogenous and exogenous parameters.

    Our response:
    Thank you! We adjusted the wording of the respective paragraph to clarify our intentions (which was not to introduce new potential independent variables, please excuse us for the misunderstanding). Also, rows 80-83 and 255-258 were adjusted to better distinguish endogenous and exogenous parameters.

13. Line 203: I would argue that this is rather an attempt to explore the association in a quantitative way rather than quanitfying the association

Our response:
Thank you! The wording has been amended accordingly.

14. Lines 205-210: as in the introduction, unclear if the contrast here is to literature on these issues in general or only literature on Hungary.

    Our response:
    Thank you! We feel that our modifications carried out in relation to earlier suggsetions cover this observation from the Reviewer.

15. 213-214: Please clarify this and link to a fundamental description of decision-making processes in Hungary.

    Our response:
    Thank you! We amended rows 286-288 to reflect on this observation.

16. Lines 232-237: this needs further explanation (what is the new law on/what does it say-how does it change the situation)

    Our response:
    Thank you! We adjusted the wording in rows 324-326. The new legislation covers expenses on IFRs of reimbursed compounds (outside of their reimbursed indications).

17. The limitations of the study need to be discussed in further detail, including the chosen methodology and the adequacy of the independent variables. Why this article is of interest beyond Hungary should be substantiated further

    Our response:
    Thank you! We amended rows 286-292 to reflect on this observation.

18. Conclusions: The recommendations in the abstract do not appear in the main text (see also comment above). The potential targets (meaning legislative change and more MEAs?) should be justified in more detail.

    Our response:
    Thank you! We feel that our modifications carried out in relation to earlier suggsetions cover this observation from the Reviewer.
